# Blood concentrations of carotenoids and retinol and lung cancer risk: an update of the WCRF–AICR systematic review of published prospective studies

**DOI:** 10.1002/cam4.676

**Published:** 2016-07-06

**Authors:** Leila Abar, Ana Rita Vieira, Dagfinn Aune, Christophe Stevens, Snieguole Vingeliene, Deborah A. Navarro Rosenblatt, Doris Chan, Darren C. Greenwood, Teresa Norat

**Affiliations:** ^1^ Department of Epidemiology and BiostatisticsImperial CollegeLondonUnited Kingdom; ^2^ Department of Public Health and General PracticeFaculty of MedicineNorwegian University of Science and TechnologyTrondheimNorway; ^3^ Biostatistics UnitCentre for Epidemiology and BiostatisticsUniversity of LeedsLeedsUnited Kingdom

**Keywords:** Carotenoids, continuous update project, lung cancer, meta‐analysis, retinol

## Abstract

Carotenoids and retinol are considered biomarkers of fruits and vegetables intake, and are of much interest because of their anti‐inflammatory and antioxidant properties; however, there is inconsistent evidence regarding their protective effects against lung cancer. We conducted a meta‐analysis of prospective studies of blood concentrations of carotenoids and retinol, and lung cancer risk. We identified relevant prospective studies published up to December 2014 by searching the PubMed and several other databases. We calculated summary estimates of lung cancer risk for the highest compared with lowest carotenoid and retinol concentrations and dose–response meta‐analyses using random effects models. We used fractional polynomial models to assess potential nonlinear relationships. Seventeen prospective studies (18 publications) including 3603 cases and 458,434 participants were included in the meta‐analysis. Blood concentrations of *α*‐carotene, *β*‐carotene, total carotenoids, and retinol were significantly inversely associated with lung cancer risk or mortality. The summary relative risk were 0.66 (95% confidence interval [CI]: 0.55–0.80) per 5 *μ*g/100 mL of *α*‐carotene (studies [*n*] = 5), 0.84 (95% CI: 0.76–0.94) per 20 *μ*g/100 mL of *β*‐carotene (*n* = 9), 0.66 (95% CI: 0.54–0.81) per 100 *μ*g/100 mL of total carotenoids (*n* = 4), and 0.81 (95% CI: 0.73–0.90) per 70 *μ*g/100 mL of retinol (*n* = 8). In stratified analysis by sex, the significant inverse associations for *β*‐carotene and retinol were observed only in men and not in women. Nonlinear associations were observed for *β*‐carotene, *β*‐cryptoxanthin, and lycopene, with stronger associations observed at lower concentrations. There were not enough data to conduct stratified analyses by smoking. In conclusion, higher blood concentrations of several carotenoids and retinol are associated with reduced lung cancer risk. Further studies in never and former smokers are needed to rule out confounding by smoking.

## Introduction

Lung cancer is the first most common cancer among men and the third most common cancer among women worldwide with 1.82 million cases and 1.59 million deaths due to lung cancer in 2012 1. The incidence rate has decreased since the mid‐1980s by 1.9% in men and the mid‐2000s by 1.2% in women. The mortality rate declined from 2004 and 2008 by 2.6% and 0.9% per year in men and women, respectively 2. Tobacco smoking accounts for more than 80% of all lung cancers 3, 4 and the increasing risk is parallel to an increases in tobacco use 2.

Diet may also play a role in lung cancer etiology 4, 5, 6. Among dietary factors, fruits, and vegetables are of much interest due to their potential anti‐inflammatory and antioxidant properties 7. Carotenoids are found predominantly in fruit and vegetables 8. Blood carotenoids have been found to be highly correlated with fruits and vegetables intake in several studies, and are considered intake biomarkers of fruit and vegetable 9, 10, 11.

According to World Cancer Research Fund/American Institute for Cancer Research (WCRF/AICR) Second Expert Report from 2007, foods containing carotenoids may protect against lung cancer (strength graded as probable) 12. By contrast, two large randomized double‐blind placebo‐controlled trials, the alpha‐tocopherol‐*β*‐carotene (ATBC) and the *β*‐carotene and Retinol Efficacy Trial (CARET) showed an increased risk of lung cancer among high‐risk people supplemented with high doses of *β*‐carotene and/or *α*‐tocopherol 13, 14, 15, 16.

A previous meta‐analysis of prospective observational studies suggested a significant inverse association between lycopene and total carotenoids and lung cancer risk, however, the number of studies on blood concentrations of carotenoids was limited and there was no exploration of the shape of the dose–response relationship between carotenoids and lung cancer 6.

More recently, two additional prospective studies have been published, including 11,003 participants and 368 lung cancer cases 4, 17. As part of the WCRF/AICR Continuous Update Project (CUP), we conducted an updated systematic review and meta‐analysis of cohort studies with the aim to clarify the relationship of blood carotenoids and lung cancer risk. Retinol was also included in this review because of the conflicting results of randomized controlled trials 13, 14, 15, 16.

## Material and Methods

### Search strategy

PubMed and several other databases, including, Embase, CABAbstracts, ISI Web of Science, BIOSIS, LILACS, Cochrane library, CINAHL, AMED, National Research Register, and In Process Medline, were searched for studies on blood concentrations of carotenoids and retinol up to January 2006 by several reviewers at the Johns Hopkins University for the WCRF/AICR Second Expert Report 12. As all the relevant studies were identified by the PubMed search, we searched the PubMed database from January 2006 up to December 2014. The specific search criteria and the review protocol can be found at http://www.wcrf.org/sites/default/files/protocol_lung_cancer.pdf. We also handsearched the reference lists of relevant articles, reviews, and meta‐analyses identified in the search.

### Study selection

Included were prospective cohort, nested case–control or case–cohorts studies that reported estimates of the relative risk (RR) (e.g., hazard ratio, risk ratio, or odds ratio) and 95% confidence intervals (CIs) of specific carotenoids, total carotenoids, or retinol in blood and lung cancer incidence or mortality. In case of multiple publications of the same study, the newest publication that included the largest number of cases was selected.

### Data extraction

The following data were extracted from each publication: first author's last name, publication year, country where the study was conducted, the study name, follow‐up period, sample size, sex, age, number of cases, laboratory method for analysis, concentrations of carotenoids or retinol, and associated RRs and 95% CIs, and variables used in adjustment in the analysis.

The search and data extraction of articles published up to December 2005 was conducted by several reviewers at the John Hopkins University during the systematic literature review for the WCRF/AICR Second Expert Report (available online: http://www.wcrf.org/sites/default/files/SLR_lung.pdf). The search and extraction from January 2006 and up to December 2014 was conducted by the CUP team at Imperial College London.

### Statistical methods

Meta‐analysis of the highest compared with the lowest blood concentrations of carotenoids and retinol, and the dose–response associations with lung cancer were conducted. Random effect models were used to calculate the summary RRs and 95% CIs to take into account heterogeneity across studies 18. Heterogeneity was determined using *Q* and *I*² statistics 19, and was explored in stratified analyses when there were eight or more studies in the analysis.

When continuous risk estimates were not provided in the articles, dose–response associations and 95% CIs were derived from categorical data using generalized least‐squares for trend estimation 20, which required the RRs and CIs associated to at least three categories of blood concentrations, number of cases, and noncases or person years of follow up per category.

The mean or median values per category were used if provided in the articles, or the midpoint was calculated for studies that only reported a range of blood concentrations of carotenoids and retinol by category. When the range of the highest or lowest category of carotenoid/retinol concentrations was open‐ended, its width was assumed to be the same as the adjacent category.

If only the total number of cases or person years was reported in the articles, and the exposure was categorized in quantiles, the distribution of cases or person years was calculated by dividing the total number of cases or person years by the number of quantiles. If the results were reported for men and women separately, they were combined using a fixed effects meta‐analysis before being pooled with other studies.

For studies that reported blood concentrations in *μ*mol/L, the units were converted to *μ*g/100 mL by dividing the concentration in *μ*mol/L by 0.01863 for *α*‐carotene, *β*‐carotene, lycopene, and total carotenoids, and by 0.01809, 0.01758, and 0.03491 for *β*‐cryptoxanthin, lutein/zeaxanthin, and retinol, respectively 21.

Small‐study effects, such as publication bias, were assessed using funnel plot and Egger's test 22.

A potential nonlinear dose–response association between blood concentrations of carotenoids and retinol was assessed using fractional polynomial model 19 and the best‐fitting second‐order fractional polynomial regression model, defined as the one with the lowest deviance was determined. A two‐tailed *P* < 0.05 was considered statistically significant.

In all analyses, the results of each paper with the most comprehensive adjustment for confounders were included. Stata version 12 software (StataCorp, College Station, TX) was used for all analyses.

## Results

**Figure 1:**
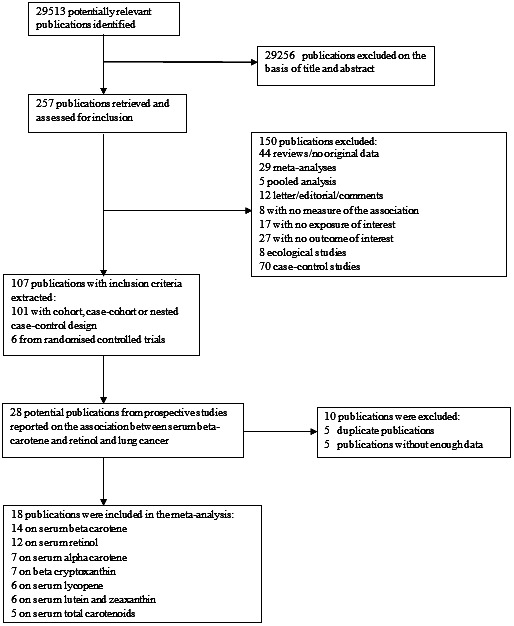
Flowchart of study selection.

From 29,513 articles identified by the search of the Continuous Update Project, 28 articles (4 during the CUP and 24 during the SLR 2005), which met the inclusion criteria were included (flowchart of study selection—Fig. 1). Ten publications were excluded; five were duplicate publications and five publications did not provide enough data for analysis. In total, 18 publications (17 cohort studies) were included in the analyses 4, 13, 17, 23, 24, 25, 26, 27, 28, 29, 30, 31, 32, 33, 34, 35, 36, 37 (Table 1). Fourteen studies (3143 cases) reported on *β*‐carotene 4, 17, 23, 24, 25, 26, 27, 28, 29, 30, 31, 32, 33, 34, seven studies on *α*‐carotene (1205 cases) 4, 13, 17, 23, 24, 25, 26 and *β*‐cryptoxanthin (1205 cases) 4, 17, 23, 24, 25, 26, 27, six studies on lycopene (1097 cases) 4, 17, 23, 24, 25, 26, lutein and zeaxanthin (927 cases) 4, 17, 23, 24, 26, 27, and five studies on total carotenoids (724 cases) 4, 23, 24, 26, 29. Twelve studies (3192 cases) were on retinol 4, 23, 24, 25, 26, 27, 28, 31, 34, 35, 36, 37. Nine publications were among men only 13, 26, 27, 28, 29, 30, 31, 33, 35 and nine publications were in both men and women 4, 17, 23, 24, 25, 32, 34, 36, 37. Eight studies were from United States, four studies from Europe, four studies from Asia, and one study from Australia (Table 1).

**Table 1:** Summary table of included studies

Author, year, country	Study characteristics	Sex Cases and controls/noncases	Exposure assessment	Exposure	Comparison	RR (95%CI)	Adjustment for confounders
Min, 2014, USA 1	NHANES III, Prospective cohort studyFU: >20 years	Men and women 161 lung cancer deaths 10,221 noncases	Isocratic HPLC methods Mean intra‐assay CV between 7 and 11%	*α*‐Carotene	≥6 vs. ≤1 *μ*g/dL	0.53 (0.32–0.88)	Age, sex, ethnicity, education, alcohol consumption, exercise, smoking status, pack‐year of smoking, obesity, total cholesterol, daily intakes of fats, vegetable and fruit consumption
				*β*‐Carotene	≥24 vs. ≤8 *μ*g/dL	0.76 (0.48–1.20)	
				*β*‐Cryptoxanthin	≥13 vs. ≤5 *μ*g/dL	0.56 (0.33–0.96)	
				Lycopene	≥29 v s. ≤13 *μ*g/dL	0.67 (0.42–1.07)	
				Lutein and zeaxanthin	≥28 vs. ≤14 *μ*g/dL	0.73 (0.44–1.22)	
Epplein, 2009, USA 2	Multi‐ethnic study, Nested Case Control, Age: 45–75 yearsMedian FU: 1 year and 8 months	Men 136 cases272 controls	Isocratic HPLC methods (94% fasting for 8 h or more)	*α*‐Carotene	100 vs. 24 ng/mL	0.24 (0.11–0.53)	Age at specimen collection, fasting hours before blood draw, cigarettes pack‐years, and pack‐years squared, years of schooling, and family history of lung cancer
				*β*‐Carotene	497 vs. 82 ng/mL	0.30 (0.15–0.64)	
				*β*‐Cryptoxanthin	353 vs. 82 ng/mL	0.33 (0.15–0.73)	
				Lycopene	463 vs. 164 ng/mL	0.36 (0.18–0.75)	
				Lutein and zeaxanthin	623 vs. 250 ng/mL	0.45 (0.21–0.94)	
				Total carotenoids	2030 vs. 908 ng/mL	0.32 (0.15–0.68)	
				Retinol	1804 vs. 890 ng/mL	1.26 (0.57–2.77)	
		Women 71 cases142 controls	Mean intra‐assay CV 4.8 to 10%	*α*‐Carotene	109 vs. 22 ng/mL	1.52 (0.53–4.38)	
				*β*‐Carotene	508 vs. 100 ng/mL	1.33 (0.49–3.61)	
				*β*‐Cryptoxanthin	413 vs. 82 ng/mL	1.58 (0.59–4.23)	
				Lycopene	401 vs. 144 ng/mL	1.94 (0.72–5.22)	
				Lutein and zeaxanthin	563 vs. 236 ng/mL	2.23 (0.79–6.26)	
				Total carotenoids	2091 vs. 818 ng/mL	1.78 (0.62–5.08)	
				Retinol	1712 vs. 777 ng/mL	0.77 (0.29–2.06)	
Alfonso, 2006, Australia 3	Wittemoon Prospective cohort study, people exposed to blue asbestos Mean age: 51.5 yearsFU: 10.5 years	Men and women 47 cases1953 noncases	Not fasting blood sample measured by HPLC	Carotene	Per 1 unit increase (*μ*mol/L)	0.41 (0.15–1.14)	Age, sex, smoking status, asbestos exposure, and level of hepatic enzymes
				Retinol		0.90 (0.54–1.51)	
Goodman, 2003, USA 4	Carotene and Retinol Efficacy Trial, Nested Case Control, High‐risk individuals aged 45–69 yearsFU: 4 years	Men and women 276 cases276 controls	Not fasting blood sample (serum stored at −70°C) HPLC, CV <10%	*α*‐Carotene	51.5 vs. 19 ng/mL	0.77 (0.45–1.32)	Age, sex, smoking, study centre at randomization, year of randomization pack‐years of smoking and years quit smoking
				*β*‐Carotene	255 vs. 87 ng/mL	1.07 (0.63–1.83)	
				*β*‐Cryptoxanthin	87 vs. 39.5 ng/mL	0.76 (0.44–1.28)	
				Lycopene	437 vs. 213 ng/mL	0.86 (0.52–1.43)	
				Lutein and zeaxanthin			
				Retinol	777 vs. 577 ng/mL	0.69 (0.42–1.14)	
Ito, 2005 (a), Japan 5	Japan Collaborative Cohort Study, Nested Case Control, Age: 40–79 yearsFU: 10 years	Men 163 cases174 controls	Serum sample (measured by HPLC and stored at −80°C for 11 years)	*α*‐Carotene	≥0.09 vs. <0.03 *μ*mol/L	0.40 (0.18–0.86)	Age, sex, smoking habits, participating institution, and alcohol drinking
				*β*‐Carotene	≥0.58 vs. <0.14 *μ*mol/L	0.23 (0.09–0.55)	
				*β*‐Cryptoxanthin	≥31 vs. <0.08 *μ*mol/L	0.32 (0.13–0.78)	
				Lycopene	≥0.15 vs. <0.04 *μ*mol/L	0.44 (0.19–1.05)	
				Lutein and zeaxanthin	≥1.15 vs. <0.64 *μ*mol/L	0.66 (0.33–1.35)	
				Total carotenoids	≥2.53 vs. <1.22 *μ*mol/L	0.42 (0.19–.95)	
				Retinol	≥3.23 vs. <2.19 *μ*mol/L	0.49 (0.22–1.08)	
		Women 48 cases112 controls		*α*‐Carotene	≥0.15 vs. <0.06 *μ*mol/L	0.39 (0.07–2.1)	
				*β*‐Carotene	≥1.21 vs. <0.40 *μ*mol/L	0.82 (0.19–3.58)	
				*β*‐Cryptoxanthin	≥0.49 vs. <0.19 *μ*mol/L	1.0 (0.22–4.48)	
				Lycopene	≥0.20 vs. <0.07 *μ*mol/L	0.63 (0.12–0.25)	
				Lutein and zeaxanthin	≥1.42 vs. <0.70 *μ*mol/L	0.29 (0.05–0.60)	
				Total carotenoids	≥3.93 vs. <1.87 *μ*mol/L	0.27 (0.06–1.34)	
				Retinol	≥2.78 vs. <1.92 *μ*mol/L	2.25 (0.68–7.47)	
Ito, 2005 (b), Japan 6	Japan, Hokkaido Cohort Study, Prospective Cohort, Age: 39–79 yearsFU: 10.5 years	Men and women 31 cases3182 noncases	Fasting serum sample, HPLC method	*α*‐Carotene	Highest vs. lowest	0.97 (0.41–2.30)	Age, sex, ALT activity, serum cholesterol, smoking habits
				*β*‐Carotene		1.55 (0.53–4.56)	
				*β*‐Cryptoxanthin		0.66 (0.18–2.36)	
				Lycopene		0.93 (0.39–2.24)	
				Lutein and zeaxanthin		1.27 (0.42–3.87)	
				Total carotenoids		1.34 (0.47–3.77)	
				Retinol		0.46 (0.14–1.50)	
Ratnasinghe, 2003, China 7	Chinese Miners, High‐risk Population Study, Nested Case Control, Age: 40–74 yearsFU: 6 years	Men 108 cases216 controls	Serum collected 2 years prior to diagnosis HPLC CV 3.2–11.4%	*β*‐Carotene	19–90 vs. <9 *μ*g/dL	2.0 (0.11–3.8)	Age, radon exposure, pack‐years smoking
				*β*‐Cryptoxanthin	>8 vs. <4 *μ*g/dL	2.9 (1.4–5.8)	
				Lycopene			
				Lutein and zeaxanthin	>61 vs. <44 *μ*g/dL	1.3 (0.7–2.4)	
				Retinol	>60 vs. <42 *μ*g/dL	0.70 (0.40–1.30)	
		
Ratnasinghe, 2000, China 8	Chinese Miners, High‐risk Population Study, Nested Case Control, Age: 40–74 years	108/216	Serum collected 2 years prior to diagnosis	*α*‐Carotene	<1 vs. >1 *μ*g/dL	1.2 (0.70–2.0)	Age, radon exposure, smoking habits, pack‐years
				*β*‐Carotene	>19 vs. <9 *μ*g/dL	2.0 (1.1–3.8)	
				*β*‐cryptoxanthin	>8 vs. <4 *μ*g/dL	2.9 (1.4–5.8)	
				Lutein and zeaxanthin	>61 vs. <44 *μ*g/dL	1.3 (0.7–2.4)	
Holick, 2002, Finland 9	Alpha‐Tocopherol, Beta‐Carotene Cancer Prevention Study, Prospective Cohort, Male smokers Age: 50–69 yearsFU: 11 years	Men 1644 cases29,133 noncases	Fasting (12 h) serum sample Isocratic HPLC	*β*‐Carotene	>290 vs. <99 *μ*g/L	0.81 (0.69–0.95)	Age, years smoked, cigarettes per day, intervention (alpha‐tocopherol and *β*‐carotene supplement) serum cholesterol
				Retinol	>684 vs. <484 *μ*g/L	0.73 (0.62–0.86)	
Yuan, 2001, China 10	Shanghai, China, Nested Case Control, Age: 45–64 yearsMen FU: 12 years	Men 209 cases622 controls	Not fasting blood sample processed within 3–4 h HPLC	*α*‐Carotene	≥1.61 vs. <0.71 *μ*g/dL	1.15 (0.62–2.15)	Age at starting to smoke, average cigarettes/day, and smoking status at the time of blood draw (nonsmoker, smoker)
				*β*‐Carotene	≥16.21 vs. <7.10 *μ*g/dL	0.74 (0.42–1.30)	
				*β*‐Cryptoxanthin	≥4.54 vs. <1.81 *μ*g/dL	0.45 (0.22–0.92)	
				Lycopene	≥4.31 vs. <1.61 *μ*g/dL	0.59 (0.31–1.14)	
				Lutein and zeaxanthin	≥40.64 vs. <24.27 *μ*g/dL	0.97 (0.55–1.71)	
				Total carotenoids	≥66.57 vs. <40.48 *μ*g/dL	0.84 (0.48–1.47)	
				Retinol	≥56.58 vs. <39.61 *μ*g/dL	0.65 (0.37–1.09)	
Eichholzer, 1996, Switzerland 11	Basel Switzerland, Prospective Cohort, Age: 20–79 yearsMen FU: 17 years	Men 87 cases2974 non cases	Fasting blood sample Fluorimetric method CV 2–5%	Retinol	<2.45 *μ*mol/L vs. higher and age >60 years	2.51 (1.24–5.08)	Age, biomarkers, smoking habits, lipids
					<2.45 *μ*mol/L vs. higher and age ≤60 years	1.36 (0.60–3.07)	
Knekt, 1993, Finland 12	Finnish Mobile Clinic Health Examination Survey, Nested Case Control, Age: ≥15 yearsFU: 9 years	Men 144 cases270 controls	HPLC, stored in −20°C analyzed 15 years after collection	*β*‐Carotene	Lowest vs. highest	0.8 (0.4–1.8) (current smokers)	Age
						2.6 (0.7–8.9) (nonsmokers)	
						1.5 (0.8–3.1) (current smokers)	
				Retinol		4.4 (0.9–21.5) (nonsmokers)	
Orentreich, 1991, USA 13	Kaiser Permanent Medical Centre, Nested Case Control, Age: 26–78 yearsFU: 8 years	Men and women 123 cases246 controls	HPLC (analyzed ~15 years later)	*β*‐Carotene	Lowest vs. highest	3.0	Sex, skin color, age, smoking status, intensity, and duration
				Retinol	1.50	
Connett, 1989, USA 14	Multiple Risk Factor Intervention Trial, Nested Case Control, Age: 35–57 yearsFU: 10 years	Men 66 lung cancer deaths131 controls	Serum sample HPLC	*β*‐Carotene	Lowest vs. highest	2.32	Age, smoking habits
					Per 10 *μ*g/dL	0.72 (0.50–1.04)	
				Retinol	Lowest vs. highest	1.84	
					Per 40 *μ*g/dL	0.65 (0.44–0.97)	
Wald, 1988, UK 15	BUPA, Nested Case Control, Age: 35–64 yearsFU: 5 years	Men 50 cases 99 controls	Serum sample HPLC	*β*‐Carotene	Lowest vs. highest	0.82	Age, duration of sample storage, smoking habit, age smoking started, amount, and type of product smoked in current smokers
Friedman, 1986, USA 16	Kaiser Permanent Medical Centre, Nested Case Control, Age: 26–78 yearsFU: 8 years	Men and women 151 cases 302 controls	HPLC	Retinol	38.1–65.5 vs. 98.7–173.3 *μ*g/dL	1.20	Sex, skin color, age, smoking status, intensity, and duration
Menkes, 1986, USA 17	Washington county Maryland, Nested Case Control FU: 5 years	Men and women 99 cases 196 controls	HPLC CV 1.8–3%	*β*‐Carotene Retinol	Lowest vs. highest	2.20 1.13	Age, sex, ethnicity/race, other, smoking habits
Nomura, 1985, USA 18	Honolulu Heart Program, Nested Case Control, Age: 45–79 yearsFU: 10 years	Men 74 cases302 controls	Not fasting blood sample	*β*‐Carotene	0–15 vs. 57.1–311.5 *μ*g/dL	2.20 (0.80–6.00)	Age, smoking habits

RR, relative risk; FU, follow‐up; HPLC, high‐performance liquid chromatography; CV, coefficients of variation.

### Blood *α*‐carotene

**Figure 2:**
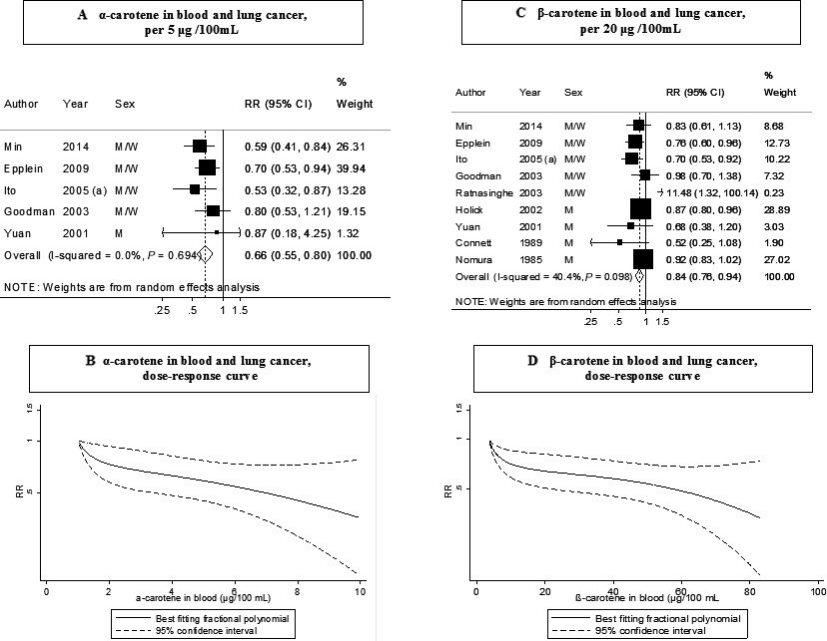
Blood concentration of *α*‐carotene (A: dose‐response analysis; B: nonlinear analysis) and *β*‐carotene (C: dose‐response analysis; D: nonlinear analysis), and lung cancer risk (dose–response and nonlinear analysis). RR, relative risk; 95% CI, 95% confidence interval. Summary RR calculated by using a random‐effects model. Ito, 2005 (a) is JACC study.

Five studies (1066 cases) were included in the dose–response meta‐analysis 4, 17, 24, 25, 26. A significant inverse association was observed (Table 2). The summary RR for an increment of 5 *μ*g/100 mL was 0.66 (95% CI: 0.55–0.80) (Fig. 2A). There was no evidence of heterogeneity (*I*² = 0%, *P*_heterogeneity_ = 0.69) or of publication or small‐study bias (*P* value Egger's test = 0.64). The overall RR for the highest versus lowest analysis was 0.70 (95% CI: 0.48–1.01, *I*² = 61%, *P*_heterogeneity_ = 0.02) in seven studies (Fig. S1A). Only three studies could be included in nonlinear meta‐analysis and no evidence of nonlinearity was observed, *P*_nonlinearity_ = 0.11 (Fig. 2B).

**Table 2:** Summary of results

Linear dose–response meta‐analysis	Studies (*n*)	Cases (*n*)	RR (95% CI)	*I*² (%)	*P*_heterogeneity_
*α*‐carotene (per 5 *μ*g/100 mL)	5	1066	0.66 (0.55–0.80)	0	0.69
*β*‐carotene (per 20 *μ*g/100 mL)	9	2958	0.84 (0.76–0.94)	40	0.10
*β*‐cryptoxanthin (per 10 *μ*g/100 mL)	6	1174	0.80 (0.57–1.12)	77	0.001
Lycopene (per 10 *μ*g/100 mL)	5	1066	0.90 (0.82–1.00)	36	0.18
Lutein and zeaxanthin (per 40 *μ*g/100 mL)	5	896	0.84 (0.66–1.07)	44	0.13
Retinol (per 70 *μ*g/100 mL)	8	2855	0.81 (0.73–0.90)	9	0.36
Total carotenoids (per 100 *μ*g/100 mL)	4	693	0.66 (0.54–0.81)	0	0.43
Highest vs. lowest meta‐analysis					
*α*‐carotene	7	1436	0.70 (0.48–1.01)	61	0.02
*β*‐carotene	14	3405	0.71 (0.56–0.91)	55	0.007
*β*‐cryptoxanthin	7	1205	0.72 (0.45–1.14)	69	0.004
Lycopene	6	1097	0.68 (0.54–0.87)	0	0.78
Lutein and zeaxanthin	6	927	0.86 (0.67–1.11)	0	0.53
Retinol	11	3145	0.72 (0.63–0.81)	0	0.91
Total carotenoids	5	724	0.64 (0.44–0.93)	23	0.27

Subgroup analysis										
Linear dose–response meta‐analysis	*β*‐carotene, per 20 *μ*g/100 mL					Retinol, per 70 *μ*g/100 mL				
	Studies (*n*)	Cases (*n*)	RR (95% CI)	*I*² (%)	*P*_heterogeneity_	Studies (*n*)	Cases (*n*)	RR (95% CI)	*I*² (%)	*P*_heterogeneity_
All studies	9	2958	0.84 (0.76–0.94)	40	0.10	8	2855	0.81 (0.73–0.90)	9	0.36
Stratified by sex										
Men	7	2466	0.80 (0.69–0.93)	63	0.01	7	2499	0.76 (0.64–0.90)	44	0.10
Women	3	221	0.69 (0.39–1.21)	7	0.34	3	221	1.01 (0.76–1.32)	0	0.50
Stratified by outcome										
Incidence	6	2518	0.88 (0.79–0.98)	43	0.12					
Mortality	3	438	0.74 (0.60–0.90)	0	0.44					
Stratified by geographic location										
USA	5	788	0.87 (0.78–0.97)	14	0.32	3	636	0.89 (0.71–1.12)	41	0.18
Europe										
Asia	3	525	0.87 (0.44–1.72)	68	0.04	3	528	0.72 (0.60–0.87)	0	0.64
After exclusion of studies in high‐risk populations										
	6	928	0.81 (0.71–0.92)	32	0.19	4	778	0.84 (0.67–1.03)	53	0.10

Subgroup analysis by blood fasting status	Studies (*n*)	Cases (*n*)	RR (95% CI)	*I*² (%)	*P*_heterogeneity_
*α*‐Carotene (per 5 *μ*g/100 mL)					
Fasting	1	207	0.70 (0.53–0.94)		
Not fasting	2	487	0.80 (0.54–1.20)	0	0.92
Not indicated	2	372	0.57 (0.43–0.77)	0	0.69
*β*‐Carotene (per 20 *μ*g/100 mL)					
Fasting	3	1959	0.86 (0.64–1.15)	70	0.04
Not fasting	3	561	0.92 (0.83–1.01)	0	0.55
Not indicated	3	438	0.74 (0.60–0.90)	0	0.44
*β*‐Cryptoxanthin (per 10 *μ*g/100 mL)					
Fasting	2	315	1.59 (0.46–5.55)	89.9	0.002
Not fasting	2	487	0.40 (0.12–1.34)	70	0.07
Not indicated	2	372	0.69 (0.55–0.87)	0	0.52
Lycopene (per 10 *μ*g/100 mL)					
Fasting	1	207	0.91 (0.80–1.03)		
Not fasting	2	487	0.78 (0.39–1.57)	46.6	0.17
Not indicated	2	372	0.71 (0.39–1.30)	69.9	0.07
Lutein and zeaxanthin (per 40 *μ*g/100 mL)					
Fasting	2	315	0.96 (0.69–1.35)	41.1	0.19
Not fasting	1	209	1.06 (0.66–1.69)		
Not indicated	2	372	0.65 (0.49–0.86)	0	0.87
Retinol (per 70 *μ*g/100 mL)					
Fasting	3	1959	0.87 (0.72–1.04)	40.8	0.18
Not fasting	3	534	0.64 (0.47–0.87)	0	0.84
Not indicated	2	362	0.80 (0.65–0.98)	21.6	0.26
Total carotenoids (per 100 *μ*g/100 mL)					
Fasting	1	207	0.74 (0.56–0.98)		
Not fasting	1	209	0.68 (0.33–1.40)		
Not indicated	2	277	0.54 (0.37–0.80)	6.6	0.30

RR, relative risk; 95% CI, 95% confidence interval.

### Blood *β*‐carotene

Nine studies (2958 cases) were included in the dose–response meta‐analysis 4, 17, 24, 25, 26, 27, 28, 29, 30. A significant inverse association was observed (Table 2). The summary RR for an increase of 20 *μ*g/100 mL was 0.84 (95% CI: 0.76–0.94) (Fig. 2C). There was moderate heterogeneity (*I*² = 40%, *P*_heterogeneity_ = 0.10) and no evidence of publication or small‐study bias (*P* value Egger's test = 0.28). An inverse association was observed in the highest versus lowest analysis (RR: 0.71; 95% CI: 0.56–0.91, *I*² = 55%, *P*_heterogeneity_ = 0.01) in 14 studies (Fig. S1B).

There was some evidence of a nonlinear dose–response of lung cancer and blood concentrations of *β*‐carotene (*P*_nonlinearity_ = 0.05, *n* = 6), with the curve showing a slightly steeper slope in the low range of *β*‐carotene concentrations (Fig. 2D), however, there was clear evidence of an inverse dose–response relationship across the range of *β*‐carotene concentrations.

### Blood *β*‐cryptoxanthin

**Figure 3:**
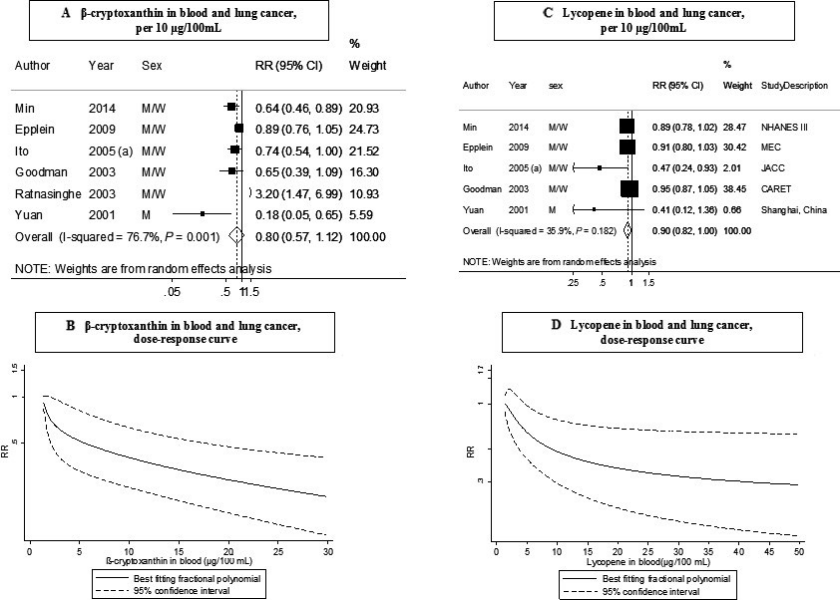
Blood concentration of *β*‐cryptoxanthin (A: dose‐response analysis; B: nonlinear analysis) and lycopene (C: dose‐response analysis; D: nonlinear analysis), and lung cancer risk (dose–response and nonlinear analysis). RR, relative risk; 95% CI, 95% confidence interval. Summary RR calculated by using a random‐effects model. Ito, 2005 (a) is JACC study.

Six studies (1174 cases) were included in the dose–response meta‐analysis 4, 17, 24, 25, 26, 27. A statistically nonsignificant, inverse association was observed (RR for an increase of 10 *μ*g/100 mL: 0.80; 95% CI: 0.57–1.12) (Table 2, Fig. 3A). There was high heterogeneity (*I*² = 77%, *P*_heterogeneity_ = 0.001). There was no evidence of publication bias with Egger's test (*P* = 0.23). Similarly, a nonsignificant inverse association was observed in the highest versus lowest analysis (RR: 0.72; 95% CI: 0.45–1.14, *I*² = 69%, *P*_heterogeneity_ = 0.004) in seven studies (Fig. S2A).

Although, the test for nonlinearity was significant (*P*_nonlinearity_ = 0.03, *n* = 4) and there was a slightly stronger association at lower blood concentrations of *β*‐cryptoxanthin, the association was nearly linear from 5 *μ*g/mL and above (Fig. 3B).

### Blood lycopene

Five studies (1066 cases) were included in the dose–response meta‐analysis 4, 17, 24, 25, 26. A borderline significant inverse association was observed (RR for an increment of 10 *μ*g/100 mL: 0.90; 95% CI: 0.82–1.00) (Table 2, Fig. 3C). There was evidence of moderate heterogeneity (*I*² = 36%, *P*_heterogeneity_ = 0.18) and publication bias (*P* value Egger's test = 0). The overall RR for the high versus low analysis was 0.68 (95% CI: 0.54–0.87, *I*² = 0%, *P*_heterogeneity_ = 0.78) in six studies (Table 2, Fig. S2B).

There was some evidence of nonlinear dose–response of lung cancer and blood concentration of lycopene (*P*_nonlinearity_ = 0.01, *n* = 3) (Fig. 3D). The inverse dose–response association appeared to be stronger at low blood concentrations of lycopene (approximately up to 20 *μ*g/100 mL) with a weaker association beyond this level.

### Blood lutein and zeaxanthin

**Figure 4:**
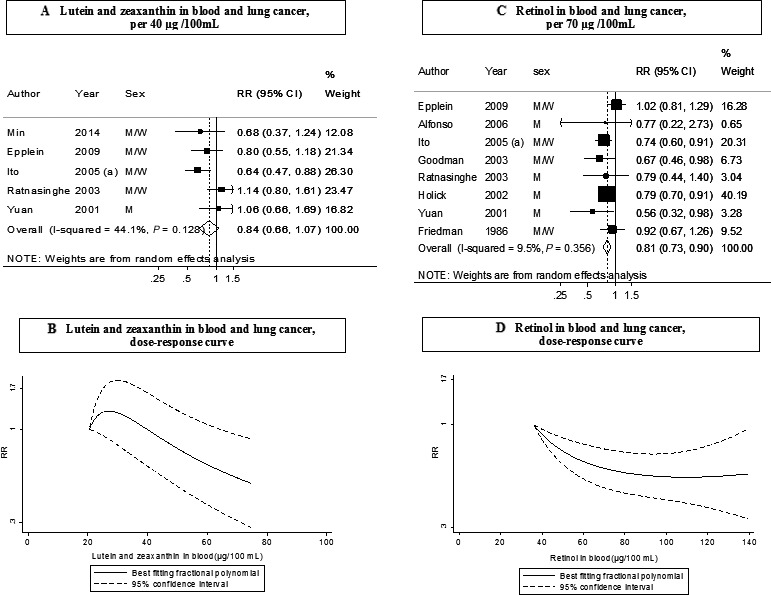
Blood concentration of lutein and zeaxanthin (A: dose‐response analysis; B: nonlinear analysis) and retinol (C: dose‐response analysis; D: nonlinear analysis), and lung cancer risk (dose–response and nonlinear analysis). RR, relative risk; 95% CI, 95% confidence interval. Summary RR calculated by using a random‐effects model. Ito, 2005 (a) is JACC study.

Five studies (896 cases) were included in the dose–response meta‐analysis 4, 17, 24, 26, 27 and six studies (927 cases) in the highest versus lowest analysis. No significant associations were observed. The summary RR for an increase of 40 *μ*g/100 mL was 0.84 (95% CI: 0.66–1.07, *I*² = 44%, *P*_heterogeneity_ = 0.13) and the RR for the highest versus lowest analysis was 0.86 (95% CI: 0.67–1.11, *I*² = 0%, *P*_heterogeneity_ = 0.53) (Table 2, Figs. 4A and S3A).

No evidence of nonlinear association was observed (*P*_nonlinearity_ = 0.51, *n* = 3) (Fig. 4B), although there was some suggestion of a negative association at higher concentrations.

### Blood total carotenoids

**Figure 5:**
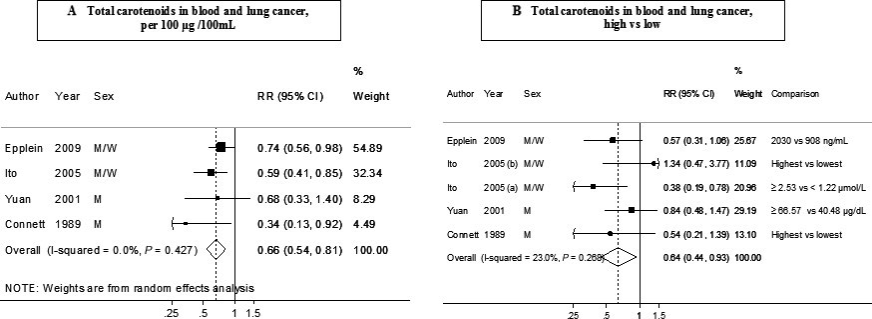
Blood concentration of total carotenoids (A: dose‐response analysis; B: high vs low analysis) and lung cancer risk (dose–response and high vs. low analysis). RR, relative risk; 95% CI, 95% confidence interval. Summary RR calculated by using a random‐effects model. Ito, 2005 (a) is JACC study and Ito, 2005 (b) is Japan, Hokkaido study.

Four studies (693 cases) were included in the dose–response meta‐analysis 4, 24, 26, 29. The summary RR for an increase of 100 *μ*g/100 mL was 0.66 (95% CI: 0.54–0.81, *I*² = 0%, *P*_heterogeneity_ = 0.43) (Fig. 5A). There was no evidence of publication bias with Egger's test (*P* = 0.30) but the number of studies was small. The overall RR for the high versus low analysis was 0.64 (95% CI: 0.44–0.93, *I*² = 23%, *P*_heterogeneity_ = 0.27) in five studies (724 cases) (Fig. 5B). The nonlinear dose–response analysis was not conducted because of the small number of studies with the required data (*n* = 2).

### Blood retinol

Eight studies (2855 cases) were included in the dose–response meta‐analysis 4, 24, 25, 26, 27, 28, 36, 37. A significant inverse association was observed. The summary RR for an increase of 70 *μ*g/100 mL was 0.81 (95% CI: 0.73–0.90, *I*² = 9%, *P*_heterogeneity_ = 0.36) (Fig. 4C). There was evidence of no publication bias (*P* = 0.67). The overall RR in high versus low analysis was 0.72 (95% CI: 0.63–0.81, *I*² =0%, *P*_heterogeneity_ = 0.91) in 11 studies (3145 cases) (Fig. S3B). There was some evidence of a nonlinear dose–response of lung cancer and serum retinol (*P*_nonlinearity_ = 0.02, *n* = 4), with wide CIs for higher exposures (Fig. 4D). No association was observed in the MEC cohort 4 in which the retinol blood concentrations were higher than in the other studies**.**

### Subgroup and sensitivity analyses

The subgroup analysis stratified by sex, cancer outcome, and geographic location was conducted only for blood *β*‐carotene and retinol because of small number of studies in the other blood carotenoids investigated. It was not possible to conduct stratified analyses by smoking status or histologic type of lung cancer because of lack of such data from the studies included.

The subgroup analysis stratified by blood fasting status was conducted and there was no strong evidence of different association as the CIs mostly overlap.

### Blood *β*‐carotene

When the analysis was repeated excluding the three studies in high‐risk populations (high‐risk miners, heavy smokers, or people exposed to asbestos) 25, 27, 28 the inverse dose–response association was slightly strengthened from 0.84 (95% CI: 0.76–0.94) to 0.81 (95% CI: 0.71–0.92) (Fig. S4A). In stratified analysis by sex, the association was significant in men (RR: 0.80; 95% CI: 0.69–0.93, *I*² = 63%, *P*_heterogeneity_ = 0.01, *n* = 7, per 20 *μ*g/100 mL) and inverse but not significant in women (RR: 0.69; 95% CI: 0.39–1.21, *I*² = 7%, *P*_heterogeneity_ = 0.34, *n* = 3, per 20 *μ*g/100 mL) for which statistical power was low (Table 2). The association was stronger in studies on lung cancer mortality (summary RR was 0.74; 95% CI: 0.60–0.90, *I*² = 0%, *P*_heterogeneity_ = 0.44, *n* = 3) than in studies on lung cancer incidence (summary RR was 0.88 (95% CI: 0.79–0.98, *I*² = 43%, *P*_heterogeneity_ = 0.12, *n* = 6), but there was no strong evidence of a difference of association as the CIs were overlapping (Table 2).

In terms of geographic location, the results were significant only in studies conducted in the United States (five studies) but not in Asia (three studies) (Table 2).

### Blood retinol

An inverse dose–response association was observed in men (2499 cases) and no association was observed in women (221 cases) (see Table 2). The summary RR's per 70 *μ*g/100 mL were 0.76 (95% CI: 0.64–0.90, *n* = 7) and 1.01 (95% CI: 0.76–1.32, *n* = 3) in men and women, respectively.

The overall estimate was no longer statistically significant when the studies in high‐risk populations were excluded 25, 27, 28, 37 (RR: 0.84; 95% CI: 0.67–1.03, per 70 *μ*g/100 mL). Only four studies remained in the analysis (Fig. S4B).

In stratified analysis by geographic location, the results were significant only in studies conducted in the Asia (three studies) but not in United States (three studies) (Table 2).

## Discussion

In this meta‐analysis, there was an inverse dose–response relationship of blood concentrations of *α*‐carotene, *β*‐carotene, and total carotenoids, and lung cancer risk. An inverse association with blood concentrations of retinol was also observed. Subjects with the highest blood concentrations of total carotenoids and retinol had 19% and 34% lower RR of lung cancer when compared to those with the lowest blood concentrations, respectively. There was little evidence of heterogeneity in these analyses. Apart from the analysis of lycopene, there was no evidence of publication bias with the statistical tests used; however, the number of studies was limited.

To our knowledge, this is the first meta‐analysis to examine a potential nonlinear association between blood concentrations of carotenoids and retinol, and lung cancer risk. The nonlinear dose–response analyses suggested inverse associations for all carotenoids, and in general, there was a stronger dose–response relationship in the lowest range of carotenoid and retinol concentrations than at the highest range. Nonlinearity was most pronounced for lycopene and retinol, for which there was a flattening of the dose–response curve at the highest concentrations, while for most of the remaining carotenoids associations were slightly stronger at lowest compared to highest concentrations, but there was a clear inverse dose–response relationship with further reductions in risk with increasing carotenoid concentrations. These findings suggests that it might be most important to avoid low blood concentrations of lycopene and retinol, and that there is little further benefit in people with highest blood concentrations, while for alpha‐carotene, beta‐carotene, and beta‐cryptoxanthin there might be further reductions in risk with increasing blood concentrations.

This study has several limitations which should be considered when interpreting the results. Smoking tends to be associated with lower intakes of fruit and vegetables, high intakes of fat and higher consumption of alcohol 38 and smokers have lower blood concentrations of some of carotenoids 39, 40, 41. Therefore, it is possible that the observed inverse associations could have been due to residual confounding by cigarette smoking. With the exception of one study that only adjusted for age 31, all the studies included in our analysis were adjusted at least for smoking status, but there was not enough data to conduct subgroup analysis by smoking status. In the only study that showed separate results in smokers and never/former smokers 17, an inverse association with lung cancer mortality was observed for *α*‐carotene and *β*‐cryptoxanthin only in current smokers but not in never/former smokers, however, in a previous meta‐analysis of fruit and vegetable intakes (some of which are high in carotenoids) and lung cancer risk, we found similar summary RRs among never smokers as compared to current or former smokers 42, although power was more limited among never smokers as the number of cases was modest.

Given the lack of data stratified by smoking status, further studies are needed in never smokers to rule out the potential confounding by smoking. Residual confounding by other factors potentially related to the blood levels of the biomarkers investigated and to lung cancer is also a possibility. When the studies in high‐risk populations—high‐risk miners, heavy smokers or people exposed to asbestos—were excluded from the meta‐analysis in sensitivity analysis, the inverse association with *β*‐carotene 25, 27, 28 was slightly strengthened from 16% to 19% and the inverse association with retinol 25, 27, 28, 37 was no longer statistically significant.

Although there was a large number of studies that could be included in the dose–response analyses of *β*‐carotene (*n* = 9) and retinol (*n* = 8), fewer studies reported on the other carotenoids (*n* = 4–6). The inverse associations were observed in men but not in women, and whether this is due to residual confounding, low number of cases in the analyses in women or gender differences is unclear and needs further study.

Furthermore, blood concentrations of carotenoids and retinol may not only reflect dietary intake, but can be influenced by the lipid content of the diet, metabolism and absorption, and genetic variability 7, 39, 40. As carotenoids and retinol are fat‐soluble, the lipid content of the diet increases the absorption. Some carotenoids including *α* and *β*‐carotene, and *β*‐cryptoxanthin can be partially metabolized to retinol, particularly in people with depleted vitamin A concentrations 40. The absorption and hence the bioavailability of carotenoids can be modulated by the fat content of the diet, competition with other carotenoids, degree of colon fermentation, and hormonal factors 40.

The results of this meta‐analysis provide further support that high blood concentrations of carotenoids and retinol, as biomarkers of fruits and vegetable intake, are associated with reduced lung cancer risk. Carotenoids are found in many different types of fruit and vegetables, and it has been shown in epidemiological studies that dietary intakes of green and raw vegetables, carrots and broccoli are correlated with blood concentrations of *α*‐carotene, *β*‐carotene, and lutein/zeaxanthin 43, and fruits and root vegetables, carrots and tomato products are good predictors of *β*‐cryptoxanthin, *α*‐carotene and lycopene in plasma 44.

In contrast to the results of many observational studies and the current meta‐analysis, two large randomized controlled trials (RCT's), the ATBC and CARET, showed an increased risk of lung cancer with high‐dose supplemental *β*‐carotene among smokers 14, 15, 16. The increased risk at high doses may be related to the prooxidant activity of *β*‐carotene when administered as a supplement in high doses (5–10 times greater than normal dietary intake) to heavy smokers 6, 45, 46. In addition, it is possible that the difference in results between the RCTs and the observational studies may be because high blood concentrations of carotenoids and retinol simply may be markers of a high fruit and vegetable intake, but may not themselves be the constituent(s) responsible for the beneficial effect. Fruits and vegetables are not only good sources of carotenoids but also contain many other vitamins, minerals, fiber, antioxidants, and numerous phytochemicals 45 that could have a potential protective effect against lung cancer, and it is possible that a number of constituents may act synergistically 47.

Strength of this meta‐analysis is the inclusion of prospective cohort studies which avoids potential recall biases and that are less prone to selection biases than case–control studies. Some analyses included a large number of cases and had statistical power to detect relatively small associations but for some micronutrients the power may have been insufficient. Most studies, as mentioned previously, were adjusted for main confounders including smoking status, intensity, duration of smoking, and other smoking variables. Most of the studies measured the carotenoids and retinol blood concentrations using high‐performance liquid chromatography (HPLC). The cancer outcome in the included studies was identified through cancer registries, death certificates and hospital records, and loss to follow‐up was very low.

In conclusion, higher blood concentrations of total carotenoids, *α*‐carotene, *β*‐carotene, lycopene, and retinol were inversely associated with lung cancer risk. However, because of the lack of data in never smokers, further large scale studies stratified by smoking status are needed to rule out residual confounding by smoking.

## Conflict of Interest

None declared.

## Supporting information


**Figure S1.** (A) *α*‐carotene in blood and lung cancer, high versus low. (B) *β*‐carotene in blood and lung cancer, high versus low.

**Figure S2.** (A) *β*‐cryptoxanthin in blood and lung cancer, high versus low. (B) Lycopene in blood and lung cancer, high versus low.

**Figure S3.** (A) Lutein and zeaxanthin in blood and lung cancer, high versus low. (B) Retinol in blood and lung cancer, high versus low.

**Figure S4.** (A) *β*‐carotene in blood and lung cancer, 20 *μ*g/100 mL. (B) Retinol in blood and lung cancer, 70 *μ*g/100 mL , after exclusion of studies in high risk populations.
