# Supplementary figures and images for: Blood concentrations of carotenoids and retinol and lung cancer risk: an update of the WCRF–AICR systematic review of published prospective studies
Source: Cancer Med. 2016 Jul 6;5(8):2069–83. doi: 10.1002/cam4.676 (PMC4971935; doi:10.1002/cam4.676)

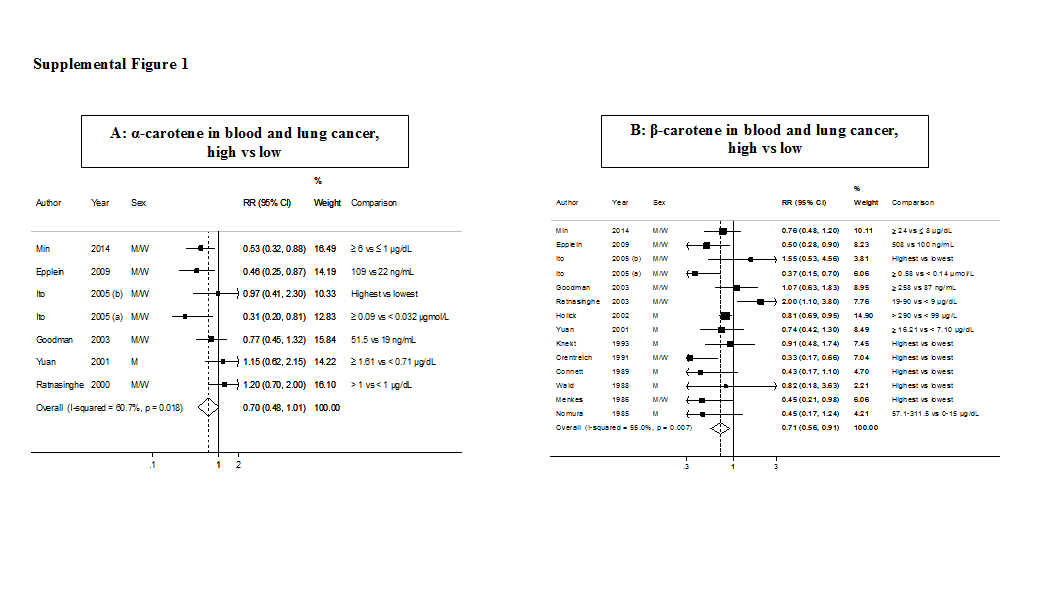

Supplement: Supplementary file 1 — Figure S1. (A) α‐carotene in blood and lung cancer, high versus low. (B) β‐carotene in blood and lung cancer, high versus low. [file CAM4-5-2069-s001.tiff]

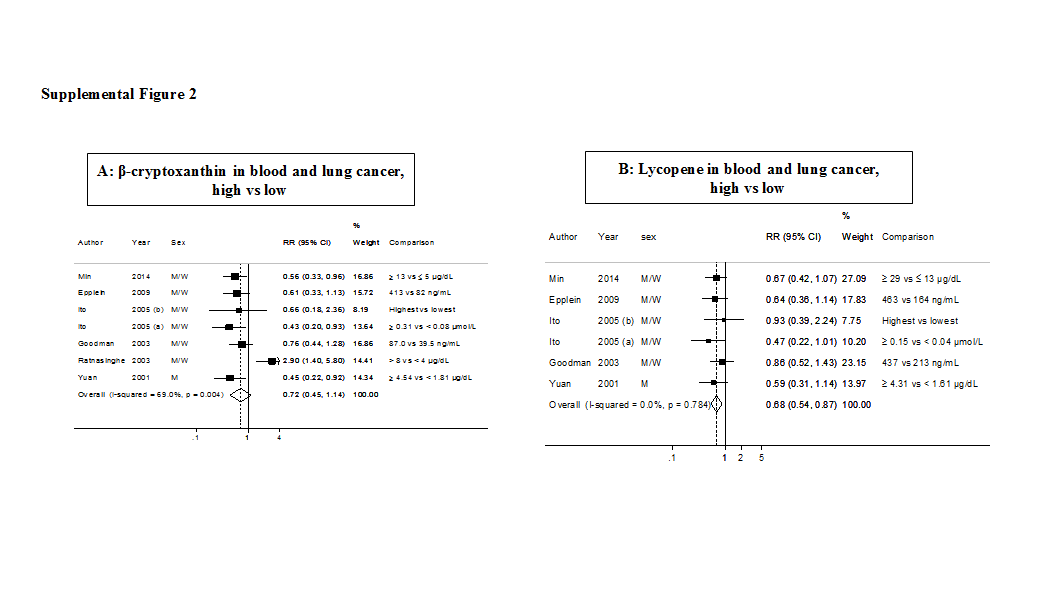

Supplement: Supplementary file 2 — Figure S2. (A) β‐cryptoxanthin in blood and lung cancer, high versus low. (B) Lycopene in blood and lung cancer, high versus low. [file CAM4-5-2069-s002.tiff]

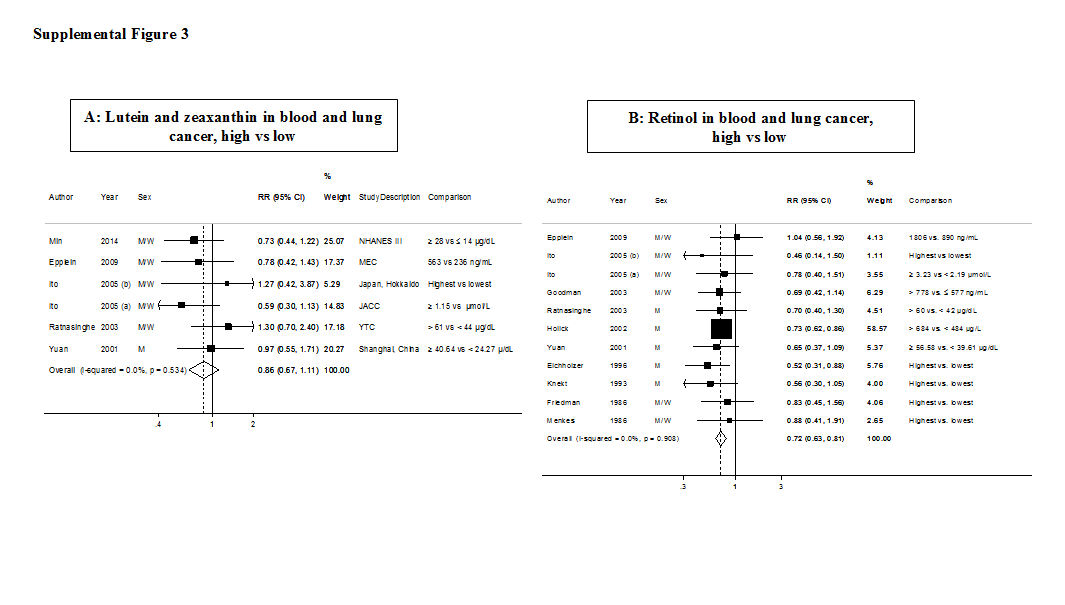

Supplement: Supplementary file 3 — Figure S3. (A) Lutein and zeaxanthin in blood and lung cancer, high versus low. (B) Retinol in blood and lung cancer, high versus low. [file CAM4-5-2069-s003.tiff]

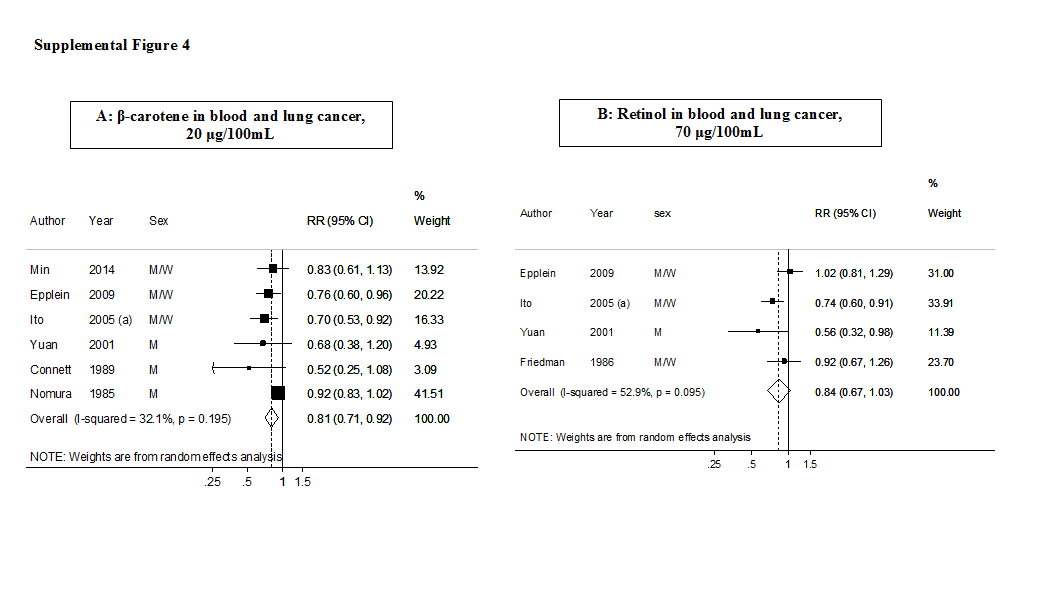

Supplement: Supplementary file 4 — Figure S4. (A) β‐carotene in blood and lung cancer, 20 μg/100 mL. (B) Retinol in blood and lung cancer, 70 μg/100 mL , after exclusion of studies in high risk populations. [file CAM4-5-2069-s004.tiff]
